# Supplementary material for: The contribution of avoidable factors in doubling or halving the odds of hypertension
Source: PLoS One. 2022 Apr 7;17(4):e0266689. doi: 10.1371/journal.pone.0266689 (PMC8989301; doi:10.1371/journal.pone.0266689)
Supplement: Attachment — Submitted filename: PONE-D-21-34725.docx [file pone.0266689.s002.docx]

Comments to the Author

This paper explored the relationship between fruit/vegetable consumption, physical activity, body mass index, waist-hip-ratio, fasting blood glucose and total cholesterol in Iran and halving or doubling the risk of hypertension. And obtained how much fruit/vegetable and physical activity halve the risk of hypertension and how much increase in body mass index, the waist-hip-ratio, fasting blood glucose, and total cholesterol can double the risk of hypertension. These results may be useful for developing guidelines. The paper would be strengthened if the following are addressed:

1. "Hypertension affects an estimated 1.13 billion people worldwide or nearly 15% of the world's population (1 in 4 men and 1 in 5 women)" in the article does not indicate the reference source.

2. Whether "fasting blood glucose, high-density lipoprotein and total cholesterol" were tested in the field or at a testing facility.

3. Whether people with hypertension were included in the group whose blood pressure was controlled through lifestyle change.

4. Which questionnaire was used for information collection, and whether the reliability and validity of the questionnaire have been verified.

5. If the detailed basic information of the study population was included in the article, such as age composition and education level, the content of the article will be fuller.

6. Since behavioral information of different ages and genders has different effects on hypertension, it is suggested to analyze the relationship between influencing factors and hypertension by stratification.

7. In Table 1, the direct incorporation of continuous data into the regression model will lead to narrow confidence interval width and small change of OR value before and after correction. It is recommended that continuous data be standardized or replaced with graded data.

8. In the discussion, there were many mechanisms to describe, and it was suggested to increase the discussion on the results in combination with the situation of Iran.

9. In the discussion of the third and sixth paragraphs, whether there are other research results similar to "consumption of every 7 servings/day of fruit and vegetable can halve the risk of hypertension" and "every 134 mg/dl increase in total cholesterol, the chance of hypertension doubles", corresponding references are suggested to be added;

10 . It is suggested to add references in recent 3 years.
