# Supplementary material for: The contribution of avoidable factors in doubling or halving the odds of hypertension
Source: PLoS One. 2022 Apr 7;17(4):e0266689. doi: 10.1371/journal.pone.0266689 (PMC8989301; doi:10.1371/journal.pone.0266689)
Supplement: Attachment — Submitted filename: Response to Reviewers.docx [file pone.0266689.s003.docx]

**Reviewer #1:**

**Introduction**

1. For clarity, the authors need to address what different forms of hypertension are.

**Answer**: We addressed this issue by adding new information to the first paragraph of the introduction section.

2. Knowing how much more exposure to a factor can halve or double the risk of an outcome of interest is critical in public health policy because it allows for better prioritization and planning of prevention programs. While it is good to know the impact of each factor on the risk of hypertension, it can be hard to have an accurate estimate due to their complex interactions. For example, fruit and vegetable consumption can affect blood pressure by influencing blood glucose, BMI, etc.

**Answer**: That is right. We can just “estimate” the impact of factors on hypertension but it is not possible to “accurately measure” the impact of a single factor on hypertension. Therefore, we added another sentence to the limitations of the study and explained this issue.

3. “At the moment, only a few studies have been conducted to determine how much exposure to these well-known factors requires to halve or double the risk of hypertension”. Please list out what factors had been examined in 2 previous studies and briefly summarize the findings.

**Answer**: We added a phrase to the introduction section and briefly summarize the findings.

**Methods**

4. “A serving size is standardized to represent 80 grams of fruit and vegetable consumption.” How were subjects instructed on the serving size? Would they need to weigh the amounts? Did they have a scale at home?

**Answer**: This survey was conducted based on the WHO STEPwise guideline. This guideline gave several examples to the participants in order to let them know how much a serving size of fruits and vegetables was. We gave some of these examples in the 4^th^ paragraph of the methods section to clarify this ambiguity.

5. “light-intensity activity is an activity that is classified as <3.5 kcal….” Would subjects know how to assess physical activities they performed as light, moderate, or vigorous? The authors need to provide more information.

**Answer**: As we explained for the previous question, this survey was performed according to the WHO STEPwise guideline. This guideline gave several examples to the participants in order to let them differentiate between low, moderate, and vigorous intensity physical activity. We gave some of these examples in the 5^th^ paragraph of the methods section to clarify this ambiguity.

**Results**

6. “The mean (SD) age of the participants was 44.50 (6.26) years with a range of 18 to 100 years” Science age is a risk factor for hypertension; more detailed information can be provided, such as median and IQR.

**Answer**: We added the median and IQR of age to the first paragraph of the results section.

7. The prevalence of hypertension was 26.3% (7,842 out of 30,542). What is the prevalence in men and women?

**Answer**: We reported the prevalence of hypertension in men and women separately in the first paragraph of the results section.

8. “Based on the multiple logistic regression analysis, current daily cigarette smoking, alcohol consumption, dairy consumption, fish consumption, and high-density lipoprotein level had no significant association with hypertension.” More data on the factors shall be presented since they were reported to associate with blood pressure.

**Answer**: We discussed about this issue in details in the discussion section.

9. “burning of 7175 kcal through physical activity at work or recreation daily.” Will this level of physical activity be practical? If not, the authors may need to use a different OR that can generate an estimate applicable for policy generation.

**Answer**: We gave an explanation in the third paragraph of the discussion section to clarify this issue.

10. “an increase in FBS of about 77 mg/dl, and an increase in total cholesterol of about 134 mg/dl.” The magnitude of FBS and TC seems not so clinically relevant. What is the OR when FBS is increased by 39 and TC by 67?

**Answer**: We added two formulas to the footnote of the Table 2 to answer this question in another way. The exposure levels of protective and risk factors that we estimated in the last column of the Table 2 are based on OR of 0.5 and 2.0, respectively. However, if someone wants to estimate the exposure levels of protective and risk factors that change the OR (for example) to 0.7 or 1.5, he/she just need to put these figures in the formula and calculate the formula to reach the answer.

**Discussion**

11. “The evidence suggests that vegetables and fruits reduce blood pressure through various mechanisms.” The authors shall need to list out the mechanisms with citations.

**Answer**: The references #22, #23, #24, and #25 (which are mentioned in the same paragraph) explain these mechanisms. We moved the above sentence to the beginning of the paragraph to remove this ambiguity.

12. “A similar study reported that every 10.3 unit increase in BMI or 50% increase in the waist-hip ratio can double the risk of hypertension “ Since the same group published the study of the reference 15, the authors need to elaborate the differences between 2 study cohorts or the data of the same cohort were used in the analysis.

**Answer**: We added additional information to the paragraph in the discussion section and explained the reason for the difference between the two studies.

13. “both of which result in renal tubular reabsorption, sodium accumulation, volume overload, and thus hypertension25-27” After this sentence, it would be great to add the information on how weight loss can decrease the risk of hypertension.

**Answer**: We added an explanation to the end of this sentence and cited it.

14. The authors need to enrich the discussion section by including more information on the factors identified in the present study and blood pressure in the literature.

**Answer**: We added some new paragraphs and several phrases to the discussion section and added six new references to the manuscript. The number of references increased from 34 to 40.

**Reviewer #2:**

This paper explored the relationship between fruit/vegetable consumption, physical activity, body mass index, waist-hip-ratio, fasting blood glucose and total cholesterol in Iran and halving or doubling the risk of hypertension. And obtained how much fruit/vegetable and physical activity halve the risk of hypertension and how much increase in body mass index, the waist-hip-ratio, fasting blood glucose, and total cholesterol can double the risk of hypertension. These results may be useful for developing guidelines.

**Answer**: Thank you.

**Reviewer #3:**

This goal of this manuscript was to explore the cut-offs associated with a given odds of having hypertension. Data appeared to have been obtained from a large survey study. The value of such information is noted in such a large scale study population. A few comments to consider.

1) The analysis performed used logistic regression which models the "odds" of an event rather than the "risk" of an event. There is a difference between "odds" and "risk". Revise to match the analysis.

**Answer**: We changed the words “risk” related to the results of this study to words “odds”.

2) The phrase "replicate the odds ratio" is not clear. Consider revising. Possibly something like the target odds ratio?

**Answer**: We paraphrased the whole main text and replaced the word “replicate” with the word “reach”.

3) The methods state that "All statistical analysis were performed at a 95% significance level...". This should likely state that all analyses were performed at the 2-sided 0.05 significance level (which corresponds to a 95% confidence level).

**Answer**: We replaced the original sentence with the suggested sentence.

4) It may be beneficial to consider rephrasing the analysis that was performed so that is clearer. The goal appear to be, at what level of some continuous variable, does the odds reach a level of 0.5 (indicating the reduction on the odds of having hypertension) and 2.0 (an increase in the odds of having hypertension). The interpretation of the coefficient of the multiple regression model is assuming other factors are held constant, for every 1-unit increase in the continuous variable, the log odds of hypertension increases by xx. For fruit and veggies, a 1 unit increase in fruit/veggie consumption, the odds of hypertension is exp(-0.09263)= 0.91. Since this is on a continuous scale, a 7 unit increase in fruit/veggie consumption corresponds to an odds ratio of exp(-0.09263*7)=0.52. There is also variability associated with this estimate as is represented by the confidence interval of the model coefficient.

**Answer**: We added the above useful and excellent explanation to end of results section.

5) The discussion section states: "The replicated OR of 0.5 or 2.0 reported here for each variable was adjusted for other model variables, in the table. In other words, we used a multiple logistic regression model accounting for all other variables. Therefore, we were able to measure the pure influence of each variable on blood pressure regardless of the other variables in the model."

However, in multiple logistic regression, each estimated coefficient is the expected change in the log odds of hypertension for a unit increase in the corresponding variables, holding the other variables constant at a certain value. Please revise this sentence with respect to the use of multiple logistic regression.

**Answer**: We replaced the original sentence with the suggested sentence.

6) Participants that were already on hypertension medication were coded to be in the "hypertension" group regardless of whether or not medication controlled their hypertension. How many subjects reported being on hypertension medication? This could potentially change the meaning of the modelled odds in that it is the odds of being hypertensive or being medicated for hypertension.

**Answer**: 22,013 out of 7,842 hypertensive patients used anti-hypertensive medications at the time of study. However, using or not using anti-hypertensive medication had no effect on the results of this study. Because, we considered hypertension as a dichotomous (0, 1) outcome in this study. If considered blood pressure as a continuous variable, using anti-hypertensive medication may affect outcome and hence the results. But it was not the case in this study.

7) To look at factors associated with increased odds of hypertension, what would happen if you did a subgroup analysis of those participants not currently medicated for hypertension? Are results consistent?

**Answer**: In this study we considered to different approaches to distinguish hypertensive patients from non-hypertensive ones. Measuring the blood pressure (as explained in the methods section) was the first approach. We also assessed participants’ history of using anti-hypertensive medication as an alternative approach to diagnose the hypertensive patients. We considered hypertension as the binomial outcome of interest in this study giving code #1 to the patients and code #0 to non-patients. Taking a history of using anti-hypertensive medication is just a diagnostic approach for increasing the sensitivity of diagnosis of the outcome. Therefore, using or not using anti-hypertensive medication had no effect on the results of this study.
