# Supplementary material for: The contribution of avoidable factors in doubling or halving the odds of hypertension
Source: PLoS One. 2022 Apr 7;17(4):e0266689. doi: 10.1371/journal.pone.0266689 (PMC8989301; doi:10.1371/journal.pone.0266689)
Supplement: Attachment — Submitted filename: Response to Reviewers.docx [file pone.0266689.s004.docx]

**Editor comments**

Please provide additional details regarding participant consent. In the Methods section, please ensure that you have specified (1) whether consent was informed and (2) what type you obtained (for instance, written or verbal). If your study included minors, state whether you obtained consent from parents or guardians. If the need for consent was waived by the ethics committee, please include this information.

**Answer**: We added the following phrase to the first paragraph in the Methods section. “This survey was a national project that was conducted for designing and implementing prevention programs against non-communicable diseases. There was no intervention in this study. Therefore, only verbal informed consent was obtained from participants. The study population included 30,542 adults aged 18 years or older. Pregnant women were excluded from the study.”

**Reviewer #2: Specific comments:**

1. "Hypertension affects an estimated 1.13 billion people worldwide or nearly 15% of the world's population (1 in 4 men and 1 in 5 women)" in the article does not indicate the reference source.

**Answer**: The reference was added.

2. Whether "fasting blood glucose, high-density lipoprotein, and total cholesterol" were tested in the field or at a testing facility.

**Answer**: Participants’ urine and blood samples were taken in the rural and urban Health Centers and then were sent to a central laboratory. We revised the main text and clarified this issue.

3. Whether people with hypertension were included in the group whose blood pressure was controlled through lifestyle change.

**Answer**: We applied different approaches to distinguish hypertensive patients from non-hypertensive ones. Measuring the blood pressure (as explained in the Methods section) was the first approach. We also assessed participants’ history of using the antihypertensive medication as an alternative approach to diagnose hypertensive patients. We considered hypertension as the binomial outcome of interest in this study giving code #1 to the patients and code #0 to non-patients. Taking a history of using anti-hypertensive medication is just a diagnostic approach for increasing the sensitivity of diagnosis of the outcome. Therefore, using or not using antihypertensive medication did not affect the results of this study.

4. Which questionnaire was used for information collection, and whether the reliability and validity of the questionnaire have been verified.

**Answer**: STEPS is the WHO's recommended tool for surveillance of NCDs and their risk factors. We used this tool to collect data and measure NCD risk factors. We added an explanation to the methods section to clarify this issue.

5. It is suggested to update the references.

**Answer**:

Vallance 1989 was replaced with Maruhashi 2013.

Clarkson 1996 was replaced with Petrie 2018.

Creager 1997 was replaced with Chen 2018.

Hayakawa 1999 was replaced with Hua 2019.

Beckman 2002 was replaced with Poznyak 2020.

Whelton 2002 was replaced with Tasnim 2020.

Chobanian 2003 was replaced with Whelton 2018.

Rice-Evans 2003 was replaced with Maaliki 2019.

Accordingly, all references are from 2005 and beyond, and about half of them are from the last 5 years.

**Reviewer #3**

All comments have been addressed and the appropriate adjustments have been made to the manuscript. No further comments.

**Answer**: Thank you.
